# Using veterinary health records at scale to investigate ageing dogs and their common issues in primary care

**DOI:** 10.1111/jsap.13809

**Published:** 2024-12-12

**Authors:** J. Jackson, A. D. Radford, Z. Belshaw, L. J. Wallis, E. Kubinyi, A. J. German, C. Westgarth

**Affiliations:** ^1^ Institute of Infection, Veterinary and Ecological Sciences University of Liverpool Neston UK; ^2^ Evivet Research Consultancy Nottingham UK; ^3^ Department of Ethology Eotvos Lorand University Budapest Hungary; ^4^ Institute of Life Course and Medical Sciences University of Liverpool Neston UK

## Abstract

**Objectives:**

The UK dog population is living longer, raising concerns about their welfare as a result of ageing‐related diseases. Our primary objective was to determine when dogs enter the “old age” life stage based on free‐text clinical narratives in veterinary electronic health records. In addition, to identify common conditions documented during consultations with old dogs.

**Materials and Methods:**

Regular expressions were developed for: ageing, elderly, geriatric, senior and old. These were used to search the veterinary clinical narratives within a large database of veterinary electronic health records. A sample of 1000 consultations were then read, and those confirmed as being old age classified according to a modified scheme based on the World Health Organization International Classification of Disease 10th Revision.

**Results:**

A total of 832 old age dogs were identified. The age at which veterinary professionals considered dogs to be in old age was over 7.25 years in 95% of patients (median age 12.5 years). This age varied among the most common breeds, with cocker spaniels being younger (median 11.7 years) compared with Jack Russell terriers (14.1 years). Weight‐related (289/832 consultations, 35%), musculoskeletal (278, 33%), dental (254, 31%), integumentary (235, 28%) and digestive (187, 22%) conditions were most common. The odds of a dental condition were greater (odds ratio: 2.71, 95% confidence interval: 1.38 to 5.31) and musculoskeletal condition less (odds ratio: 0.36, 95% confidence interval: 0.17 to 0.81) in cocker spaniels than in a mixed breeds reference.

**Clinical Significance:**

This metric applied at scale to identify old patients may provide a novel foundation for timely health interventions targeted to dogs at increased risk of developing various age‐related conditions.

## INTRODUCTION

Older dogs are estimated to account for 30% to 40% of patients seen in veterinary practice (Metzger, 2005). Their health can be negatively impacted by a wide variety of age‐associated issues affecting all body systems; as a result, the healthcare needs of dogs change as they age (Nam et al., 2024; Neilson et al., 2001; Willems et al., 2017). In previous studies, four life stages have been suggested for dogs: puppy, adult, senior and geriatric (Creevy et al., 2019; Fortney, 2012); however, the ages of transition between each of these stages, and how this varies between breeds of dogs are less well defined, limiting opportunities for more tailored veterinary care. Dogs entering the “senior” life stage are generally considered to be relatively healthy with approximately a quarter of their expected lifespan remaining (Creevy et al., 2019). In contrast, during the final “geriatric” life stage, there is a greater prevalence of age‐associated comorbidities (Creevy et al., 2019; Fortney, 2012). Variables such as breed and body weight are frequently considered when defining the progression between life stages, with larger dogs considered to reach geriatric stages some 5 years before the smallest dogs (Willems et al., 2017). However, this approach has been criticised because it might obscure breed‐specific welfare issues meaning that early mortality in large breeds is ignored (Harvey, 2021). Sex is known to affect life expectancy in humans, with women living longer than men on average (Ginter & Simko, 2013). In dogs, less is known about the impact of sex and neuter status on ageing. Based on the age of recorded death, sexually intact dogs had the shortest life expectancy (O'Neill et al., 2013), whilst in a separate study based on owner questionnaires, neutered females lived longer than males or intact females, although among dogs dying of natural causes entire females lived slightly longer (Michell, 1999). More recently, McMillan et al. (2024) have also evidence this female “survival advantage.” It is unclear whether such observed impacts on lifespan are a direct result of neutering, or whether neutering might be an indicator of owner attitudes to veterinary care, including timing of euthanasia.

Understanding the characteristics of healthy ageing and differentiating this from adverse effects of age‐associated diseases could improve the management of dogs in the senior and geriatric stages of life (Bellows et al., 2015). O'Neill et al. (2021) utilised electronic health records (EHRs) from 784 UK‐based veterinary clinics to identify common issues in dogs including disorders of the teeth (14%), skin (13%), intestine (10%), musculoskeletal system (9%), ears (8%), as well as obesity (7%); dogs aged over 9 years were more commonly diagnosed with degenerative disorders such as osteoarthritis. In contrast, owners attending first opinion practice identified sleeping more, loss of hearing and vision, increased stiffness, thirst and urination, and dental disease as important age‐related issues (Davies, 2012). However, the same study showed that owners may not be aware of, or recognise, the importance of individual signs such that in a prospective health screen of dogs over 9 years of age at least one issue was recorded in dogs' EHRs that the owner had not noticed, (Diez et al., 2015). In contrast, veterinary professionals may identify these diseases during routine consultations that could become part of a preventive screening programme/frailty instruments (Melvin et al., 2023; Robinson et al., 2016), allowing healthcare interventions to be applied in a timely manner to maintain quality of life (Davies, 2012). This emphasises the need for veterinary professionals to educate their pet owners on the signs of age‐related diseases, and reinforces the need for regular check‐ups of old age dogs (OADs) to ensure any pathological signs are detected to allow for timely medical intervention (Wallis et al., 2023).

The first aim of this study was to develop a novel text‐based approach to determine when a veterinary professional indicated that a dog was of old age, based on clinical text narratives within EHRs. After identifying a cohort of such OADs, the study also aimed to investigate how their description varied by dog age and breed. A third aim was to identify, categorise and investigate common issues reported and recorded within a sample of EHRs from OADs; the top five disease conditions were then examined for evidence they were associated with a dog's age, sex, neuter status and breed.

## MATERIALS AND METHODS

We explored a novel approach to determine the age at which dogs enter the old age life stage by examining the use of five different old age‐related terms (“ageing,” “elderly,” “geriatric,” “old” and “senior”) by veterinarians in the electronic health record (EHRs) of their canine patients. These words were chosen because of their previous use in veterinary literature (Creevy et al., 2019; O'Neill et al., 2013) and from the opinions of the authors; the phrase “old age” is used throughout this paper to refer to a dog described with any one of these five key search words.

The study population was derived from dogs whose EHRs were collected by the Small Animal Surveillance Network (SAVSNET), a sentinel network of over 500 UK veterinary clinics. At the time the study was conducted, the SAVSNET database contained EHRs from 5.1million canine consultations collected between March 2014 and November 2020 (SAVSNET 2020). Of relevance to this study, each EHR includes a free‐text narrative written by the attending veterinarian, along with age, sex, neuter status, species, breed, owner postcode and any treatments prescribed. Animals described as breed “cross,” “crossbreed” and, for example, “colliex” were grouped in a “mixed breed” category. The most common designer crossbreeds were kept as separate breeds; at the time of writing this was cockapoo. However, since they are a relatively new phenomenon they were rare in the old age cohort (*n* = 3). Provided that the owner does not opt out, these data are sent to the University of Liverpool for curation and analyses. The processes governing collection and use of data are approved by the University of Liverpool Research ethics committee (RETH000964).

Regular expressions (regex) were developed to recognise each of the old age‐related terms in Python programming language using bespoke SAVSNET software (Datalab), which also prefilters each narrative to remove most occasional potential identifiers. Regular expressions were designed in an iterative fashion to exclude any common negations; for example, a negative look forward such as “?!owner” excludes consultations relating to an elderly owner rather than an elderly dog. Datalab also includes a machine‐learning application that suggests common related phrases or spelling variations which could be incorporated into a regex to minimise the exclusion of relevant EHRs; for example, identified spelling variations of the keyword senior were included as “(senior|senoir|seniour).” The final regexes are available in Table S1. To determine their positive predictive value, the primary researcher read a random sample of 100 clinical narratives that had been identified by each regex. The finalised regexes were then applied to the full dataset including 5.1million canine consultations; identified canine consultations were exported, encrypted and stored in electronic spreadsheets (Excel Professional Plus, Microsoft Corporation 2019).

To explore possible reasons for a veterinarian using each old age term, random samples of 200 canine consultations were extracted for each regex. The number of records selected was chosen pragmatically, based on the time required for the primary researcher to read each EHR within the study timeframe. After reading, the dataset was cleaned by removing false positives (i.e., consultations which had been detected by the regex but were not deemed to be from OADs), consultations with missing data fields (age, sex, neuter status, breed) and duplicated records (when identified by more than one regex). For analysis, breeds with ≤10 consultations were combined into an “other” category; this category comprised 170 consultations.

### Identifying common old age issues and discussion points

The primary researcher read the clinical narrative to identify conditions recorded by the veterinarian. Relevant short “text fragments” were highlighted and coded using a modified version of the World Health Organization (WHO) International Statistical Classification of Diseases and Related Health Problems (ICD) ICD‐10 system (WHO, 2010). Modifications to this system included adding travel‐related conditions, microchipping, behaviour, weight, euthanasia and other conditions (see Table S2). If the appropriate classification for a text fragment was unclear, a second researcher reviewed the text and a final classification made by consensus.

In the second stage of categorisation, the free‐text fragments highlighted in the first stage were further clustered manually and iteratively into sub‐categories; in contrast to the deductive ICD‐10 categories, these sub‐categories were generated inductively in response to the language the attending veterinarian used in the EHR, rather than being imposed from a published ontology. For example, where a veterinarian had written “vomiting twice yesterday,” the sub‐category “vomiting” was created and associated with the “digestive” modified ICD‐10 category. Further, more than one condition could be identified within each clinical narrative including different conditions belonging to the same ICD‐10 category. For example, if a veterinarian recorded “lameness, mild gingivitis and tartar,” this would create three subcategories (tartar, gingivitis, lameness) but only two ICD‐10 categories (dental, musculoskeletal). Where there was a single suspected diagnosis for a dog, this was categorised; however, if there were multiple suspected diagnoses for the presenting complaint only the presenting complaint was coded. For example if “haemorrhagic diarrhoea ?salmonella ?parvovirus” was written, only haemorrhagic diarrhoea was categorised.

### Statistical analysis

Pairwise associations between the continuous variable of age and categorical variables (old age regex, breed, modified ICD‐10 category) were conducted using the Kruskal‐Wallis test, with post‐hoc comparisons made using Dunn's tests. The frequency of dogs recorded within the top five modified ICD‐10 categories was compared for each old age regex using a chi‐squared test. Univariable and multivariable binary logistic modelling was then performed to investigate factors associated with ICD‐10 categorisation outcomes (for the top five ICD10 categories: digestive, dental, integument, musculoskeletal and weight), using fixed effects of age, old age regex, breed, and sex and neuter status combined. Statistical significance was set at P ≤ 0.05 (two‐sided) and the analysis conducted using SPSS version 26.0, IBM Corp. Violin plots were constructed using online open‐access statistical package R version 4.0.2 (R Core Team, 2020) with the ggplot2 package (v3.4.2; Wickham, 2016).

## RESULTS

### Identifying old age dogs

The total number of matches for each old age regex in the entire SAVSNET database of 5.1 million canine consultations were: “old” (34,226; 6.67 per 1000 canine consultations), “senior” (23,121; 4.51 per 1000), “elderly” (6536; 1.27 per 1000), “geriatric” (3374; 0.66 per 1000) and “ageing” (1890; 0.37 per 1000). The positive predictive value of each old age regex, based on reading 100 narratives, was: “ageing” (97%), “elderly” (90%), “geriatric” (99%), “old” (52%) and “senior” (99%). Common reasons for failure of the regex are shown in Table S1; a frequent reason for error was when the term was used to describe another animal in the household, and for “old,” descriptions of old inanimate objects such as socks and medicines.

From the sample of 200 random consultations extracted for each old age regex, 169 consultations were excluded comprising 157 which were false positives, 11 which met the case definition but were missing breed data and one duplicate. The records of a further three dogs were duplicated because they had been identified by two different old age regular expressions; in these dogs, one consultation for each was randomly excluded. Therefore, the final dataset (the OAD sample) comprised 832 consultations including 176 “ageing” (21%), 164 “elderly” (20%), 197 “geriatric” (24%), 105 “old” (13%) and 190 “senior” (23%). Four hundred and twenty‐seven dogs (51%) in this sample were female (329 neutered; 77%), with the remaining 405 dogs (49%) being male (285 neutered, 70%). In total, 86 different breeds were represented (median 2 dogs per breed, range 1 to 180), the most common of which were mixed breed (180; 22%), Labrador retriever (108, 13%), Jack Russell terrier (60, 7%), cocker spaniel (48, 6%), Border Collie (47, 6%) and springer spaniel (36, 4%).

**FIG 1:**
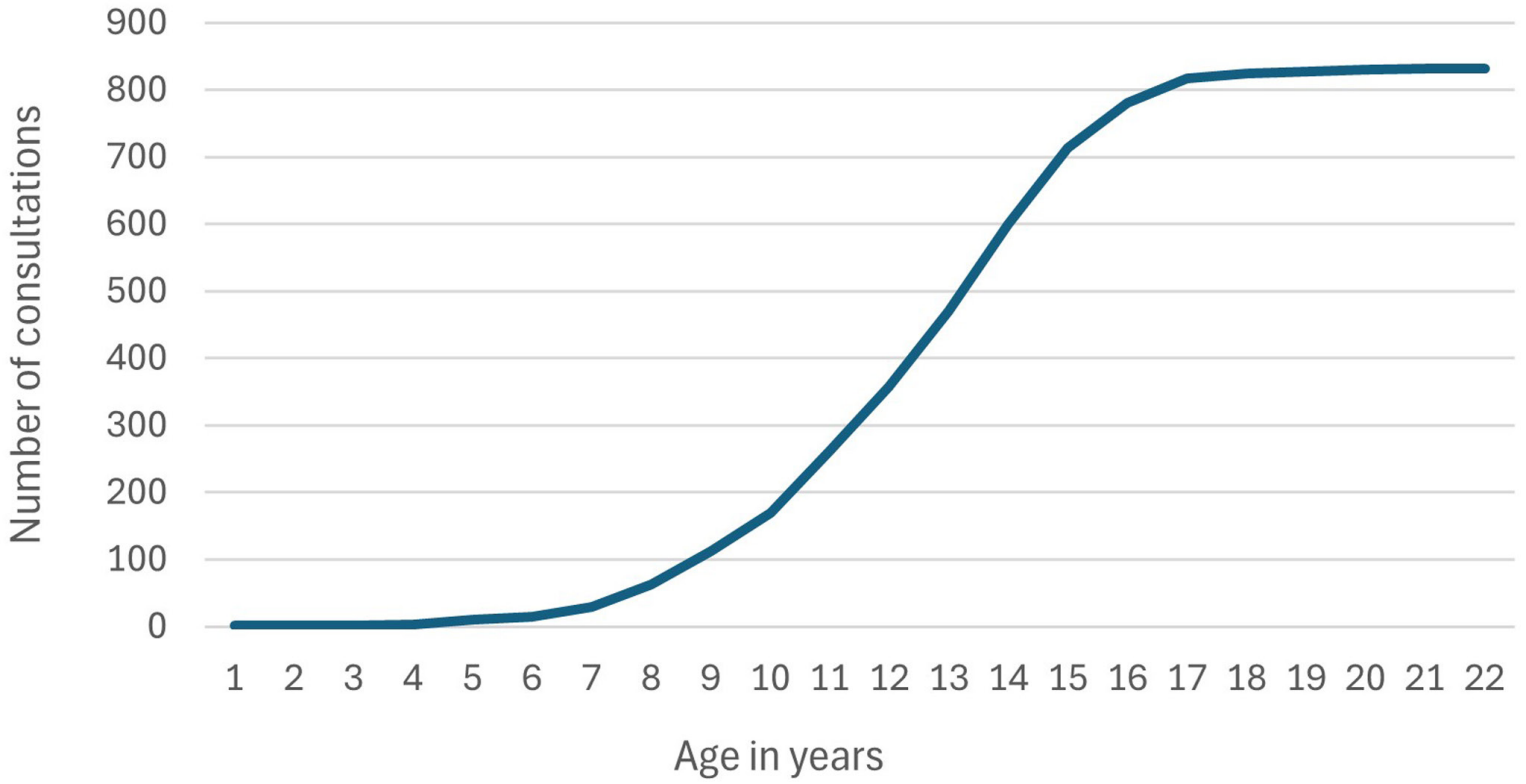
Cumulative count of the age in the 832 old age dogs used as the study population; 95% of dogs in this population were aged ≥7.25 years. For clarity, x‐axis label category “1” includes dogs aged 0 to 1 year.

The median age of the entire sample of 832 OADs was 12.5 years (range 0.0 to 20.5 years, IQR = 10.4 to 14.2), with 95% being 7.25 years of age or over (Fig 1). The younger dogs identified by the regex often reflected inaccuracies in the age field of the EHR; for example, one dog had a recorded age of zero, but the clinical narrative stated the age was unknown because it was adopted, and the attending practitioner thought the dog was actually approximately 15 years old.

**FIG 2:**
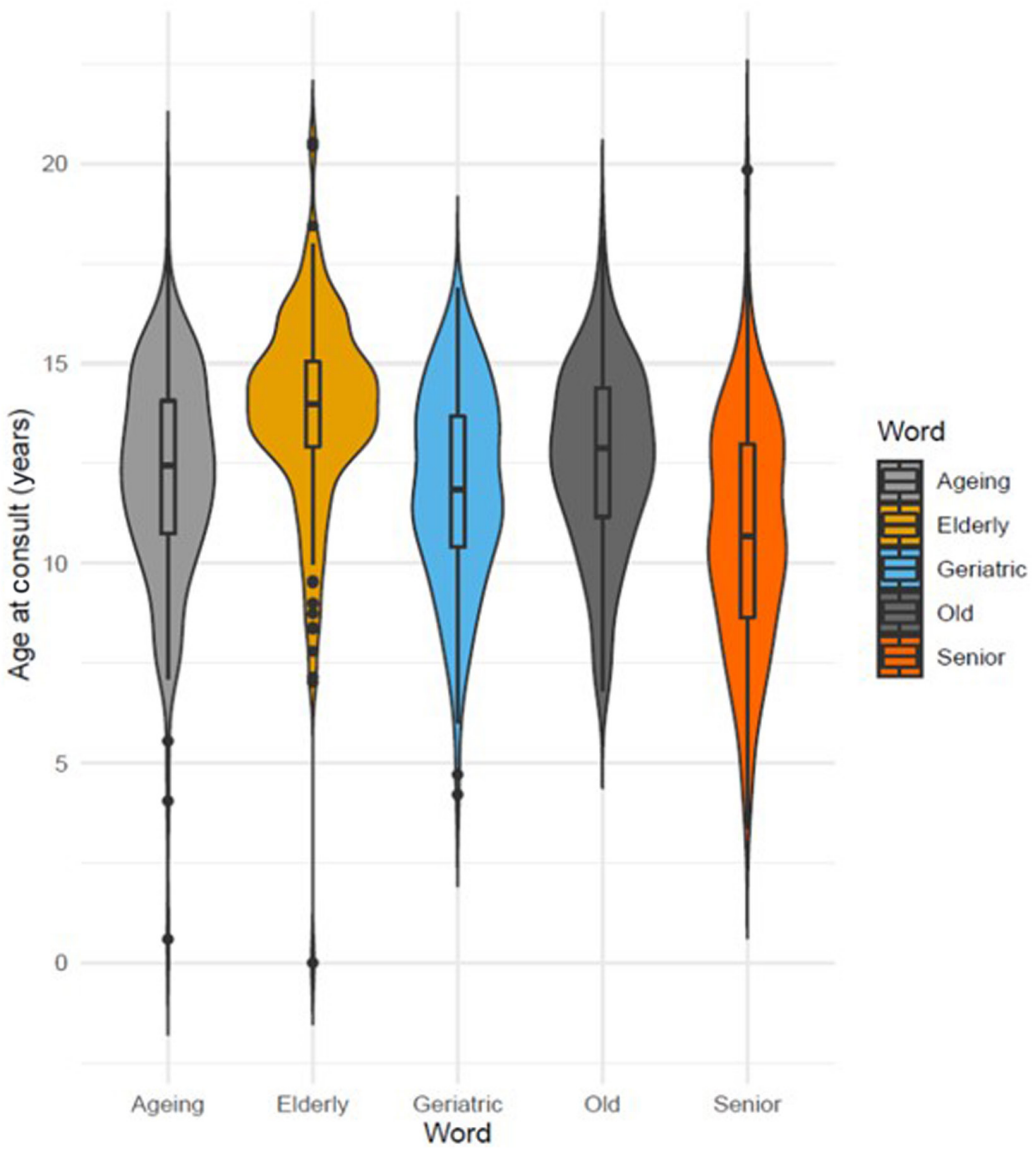
Violin plot demonstrating the median age, interquartile range and distribution along with associated data density for each of the five old age regular expressions used to identify words used by veterinary professionals to describe a dog as having reached old age in the old age dog sample (total *n* = 832).

The median age and IQR at consultation (Fig 2) for each old age regex was: “elderly” (13.9 years; 12.6 to 15.1 years), “old” (12.9 years; 11.1 to 14.4 years), “ageing” (12.3 years; 10.6 to 14.1 years), “geriatric” (11.8 years; 10.4 to 13.6 years) and “senior” (10.7 years; 8.6 to 13.0 years) (Kruskal‐Wallis P < 0.001). Post hoc Dunns test analyses showed that the age of dogs identified by the “senior” regex were younger than dogs in all of the other categories (P < 0.001), whilst dogs identified by the “geriatric” regex were younger than those identified by both the “old” (P = 0.042) and “elderly” regex (P < 0.001); finally, dogs identified by the “elderly” regex were older than dogs identified by both the “ageing” and “old” regex (P < 0.001).

**FIG 3:**
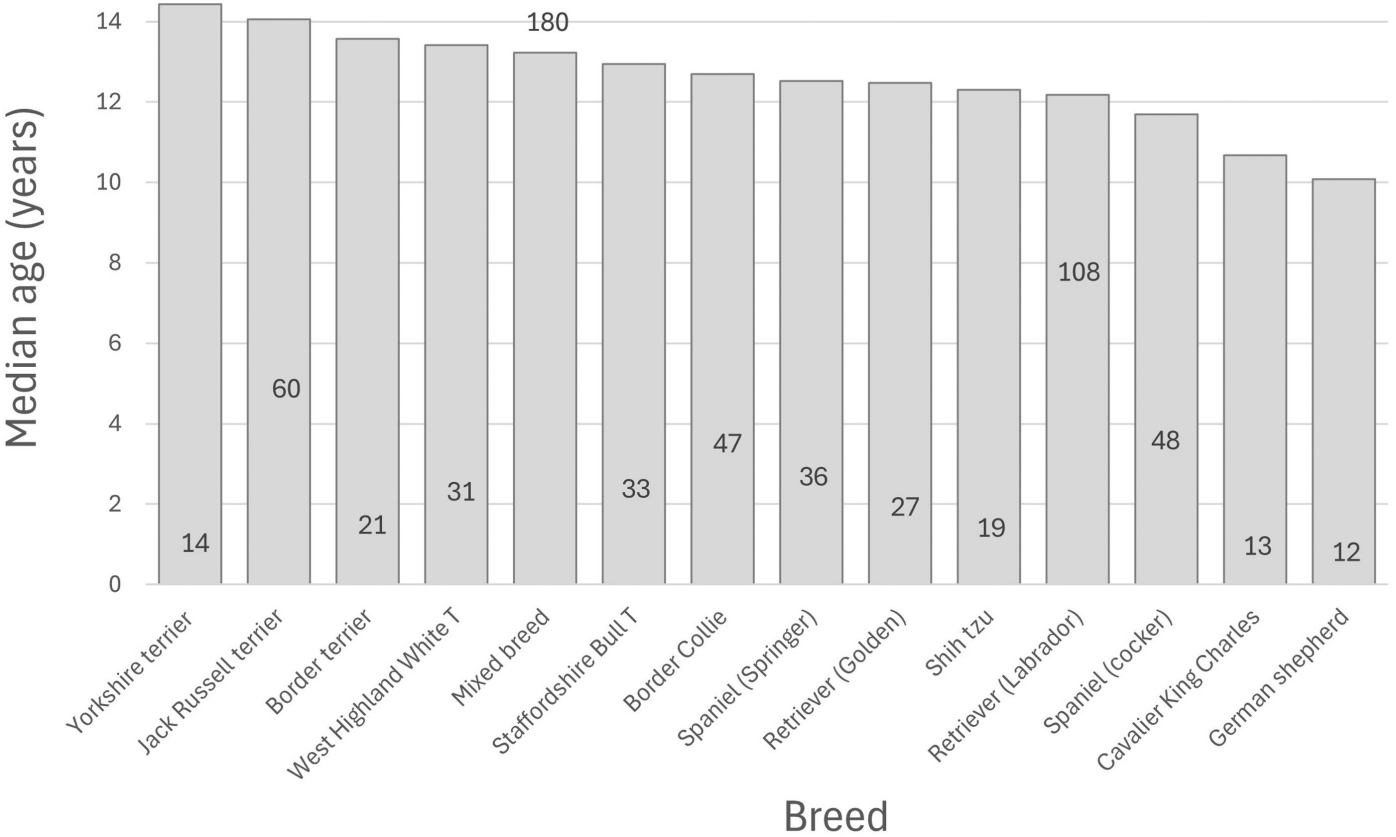
Median age (histogram) and number of consultations of given breeds with more than 10 dogs. The vertical position of each number is proportionate to the number itself.

The median age and IQR of the six most common breeds including “other breeds” were: Jack Russell Terrier (14.1 years; 10.5 to 15.9 years); mixed breed (13.2 years; 10.6 to 14.5 years); Border Collie (12.7 years; 11.3 to 14.1 years); springer spaniel (12.5 years; 10.9 to 13.8 years); Labrador retriever (12.1 years; 10.5 to 13.6 years); other breed (12.3 years; 10.1 to 13.9 years) and cocker spaniel (11.7 years; 10.2 to 13.1 years) (Kruskal‐Wallis P < 0.001) (Fig 3). Post hoc Dunns test analyses showed that Jack Russell terriers were significantly older than both cocker spaniels (P < 0.001) and Labrador retrievers (P < 0.001). The median age of the OAD sub‐sample did not significantly differ by sex and neuter status; sexually intact females (12.9 years; 10.8 to 14.3 years), neutered females (12.9 years; 10.3 to 14.4 years), sexually intact males (12.5 years; 10.5 to 14.3 years) and neutered males (12.5 years; 10.3 to 13.8 years) (P = 0.285).

### Identified common issues

**FIG 4:**
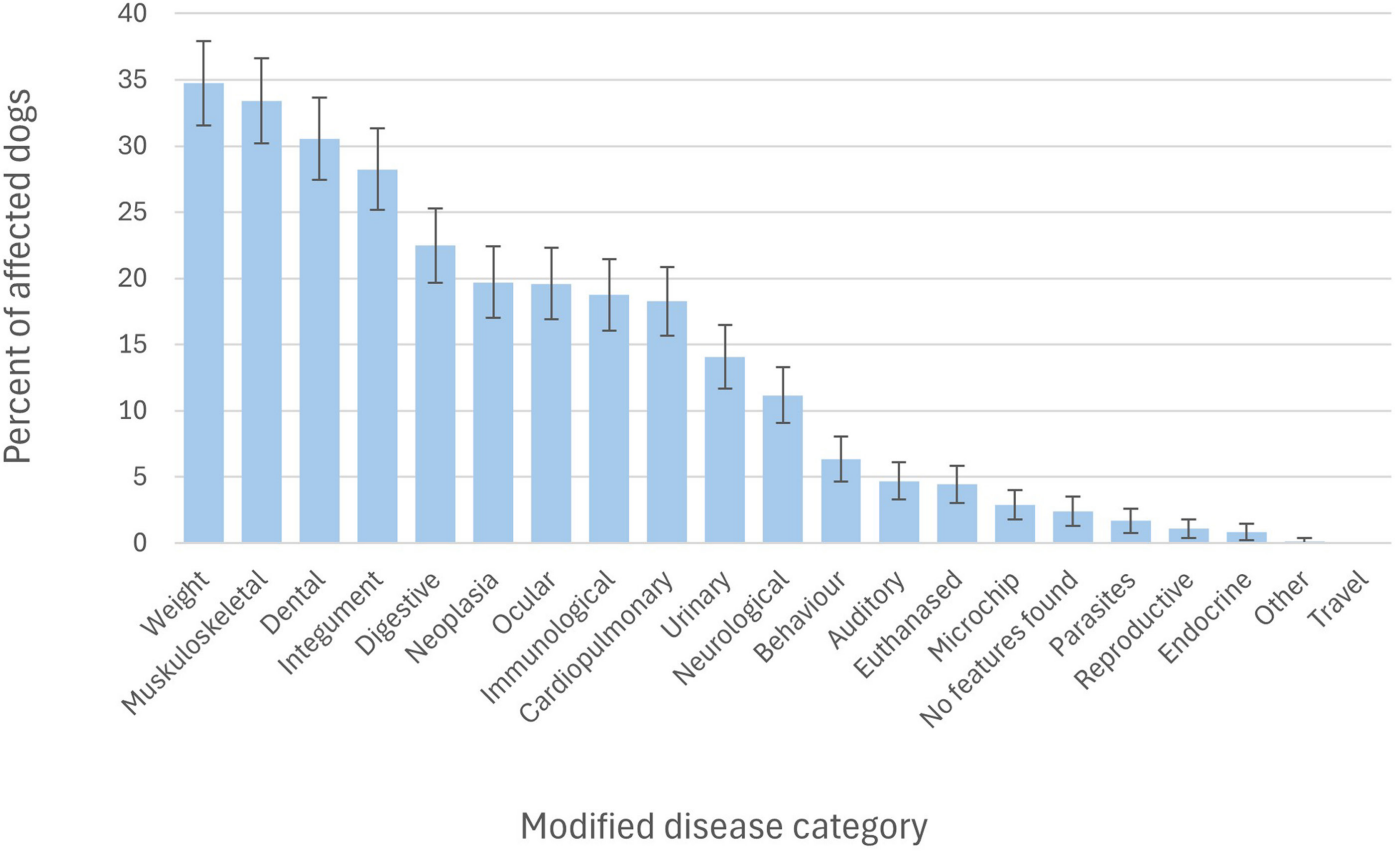
Percentage (with 95% confidence intervals) of 832 dogs affected by each of the modified ICD‐10 categories.

Within the 832 study dogs, 2944 recorded clinical issues were identified, representing a total of 2292 ICD‐10 categories (median 2, range 0 to 10 ICD‐10 categories per consultation). The five most frequent ICD‐10 categories were weight (457 clinical issues affecting 289 of the 832 individual patients; 35%), musculoskeletal (375 issues affecting 278 of 832 patients; 33.4%), dental (362 issues affecting 254 of 832 patients; 30.5%), integument (270 issues affecting 235 of 832 patients; 28.2%) and digestive (230 issues affecting 187 of 832 patients; 22.5%) (Fig 4). Across all categories (Table S3), the most common sub‐categories were tartar (109 of 2944 issues; 3.7%), weight loss (93; 3.2%), vaccination (66; 2.2%), stiffness (60; 2.0%) and murmur (57; 1.9%).

### Logistic regression modelling of ICD‐10 categorisation

Multivariable models with significant findings for each of the five most frequent sub‐category outcomes are presented in Tables 1, 2, 3, 4.

**Table 1:** Dental conditions. Univariable (unadjusted) and multivariable logistic regression analyses of variables associated with the dental modified ICD‐10 categorisation in the old age dog sample

Variable	Dental disease present	Dental disease absent	Unadjusted		Multivariable model	
	*N* (%)		OR (95% CI)	P	OR (95% CI)	P
Old age regex						
Ageing (reference)	53 (21.3)	123 (69.9)	1.00	0.99	1.00	0.58
Elderly	51 (31.1)	113 (68.9)	1.05 (0.66 to 1.66)	0.84	0.89 (0.54 to 1.43)	0.60
Geriatric	59 (29.9)	138 (70.1)	0.99 (0.64 to 1.55)	0.97	1.08 (0.69 to 1.70)	0.73
Old	31 (29.5)	74 (70.5)	0.97 (0.57 to 1.65)	0.92	1.01 (0.59 to 1.73)	0.98
Senior	60 (31.6)	130 (68.4)	1.07 (0.69 to 1.67)	0.76	1.32 (1.06 to 1.21)	0.23
Age	Median years					
	13.0	12.3	**1.10** (1.04 to 1.17)	**<0.01**	**1.13** (1.07 to 1.21)	**<0.01**
Breed	*N* (%)					
Mixed breed (reference)	49 (27.2)	131 (72.8)	1.00	0.06	1.00	0.08
Labrador retriever	31 (28.7)	77 (71.3)	1.08 (0.63 to 1.83)	0.79	1.19 (0.69 to 2.04)	0.53
Jack Russell terrier	25 (41.7)	35 (58.3)	1.91 (1.04 to 3.51)	0.04	1.72 (0.92 to 3.22)	0.09
Cocker spaniel	23 (47.9)	25 (52.1)	**2.46** (1.28 to 4.73)	**0.01**	**2.71** (1.38 to 5.31)	**<0.01**
Border collie	13 (27.7)	34 (72.3)	1.022 (0.50 to 2.10)	0.95	1.07 (0.52 to 2.21)	0.86
Springer spaniel	9 (25.7)	26 (74.3)	0.89 (0.39 to 2.03)	0.78	0.88 (0.38 to 2.03)	0.77
Other	104 (29.5)	249 (70.5)	1.12 (0.75 to 1.67)	0.59	1.19 (0.79 to 1.80)	0.39
Sex and neuter status						
Intact male (reference)	40 (33.3)	80 (66.7)	**1.00**	**0.05**	**1.00**	**0.05**
Male neutered	100 (35.1)	185 (64.9)	1.08 (0.69 to 1.70)	0.73	1.49 (0.93 to 2.37)	0.10
Intact female	31 (31.6)	67 (68.4)	0.92 (0.52 to 1.64)	0.79	**1.64** (1.15 to 2.34)	**0.01**
Female neutered	83 (25.2)	246 (74.8)	0.67 (0.43 to 1.06)	0.09	1.34 (0.81 to 2.22)	0.25

OR Odds ratio, CI Confidence interval

Model: *N* = 832 old age dogs identified by one of five regular expressions (regex). Hosmer‐Lemeshow = 0.296. Adjusted model for age, old age regex, breed, sex and neuter status. The significance of P < 0.05 values are mentioned in bold.

**Table 2:** Digestive conditions. Univariable (unadjusted) and multivariable logistic regression analyses of factors associated with digestive modified ICD‐10 categorisation in the old age dog sample

Variable	Digestive disease present	Digestive disease absent	Unadjusted		Multivariable model	
	*N* (%)		OR (95% CI)	P	OR (95% CI)	P
Old age regex						
Ageing (reference)	21 (11.9)	155 (88.1)	**1.00**	**<0.01**	**1.00**	**<0.01**
Elderly	42 (25.6)	122 (74.4)	2.54 (1.43 to 4.52)	**<0.01**	**2.35** (1.31 to 4.24)	**<0.01**
Geriatric	39 (19.8)	158 (80.2)	1.82 (1.02 to 3.24)	**0.04**	**1.86** (1.04 to 3.33)	**0.04**
Old	61 (32.1)	129 (67.9)	2.19 (1.15 to 4.17)	**0.02**	**2.22** (1.16 to 4.26)	**0.02**
Senior	24 (22.9)	81 (77.1)	**3.49** (2.02 to 6.04)	**<0.01**	**3.74** (2.13 to 6.56)	**<0.01**
Age	Median years					
	12.9	12.4	1.03 (0.97 to 1.09)	0.32	1.06 (0.99 to 1.13)	0.10
Breed	*N* (%)					
Mixed breed (reference)	40 (22.2)	140 (77.8)	1.00	0.68	1.00	0.77
Labrador retriever	17 (15.7)	91 (84.3)	0.65 (0.35 to 1.22)	0.18	0.73 (0.39 to 1.39)	0.35
Jack Russell terrier	15 (25.0)	45 (75.0)	1.17 (0.59 to 2.31)	0.66	1.17 (0.58 to 2.37)	0.65
Cocker spaniel	11 (22.9)	37 (77.1)	1.04 (0.49 to 2.22)	0.92	1.16 (0.530 to 2.54)	0.71
Border collie	12 (25.5)	35 (74.5)	1.20 (0.57 to 2.52)	0.63	1.34 (0.63 to 2.87)	0.45
Springer spaniel	7 (19.4)	29 (80.6)	0.84 (0.34 to 2.07)	0.71	0.88 (0.35 to 2.21)	0.79
Other	187 (22.5)	645 (77.5)	1.11 (0.72 to 1.70)	0.63	1.16 (0.75 to 1.80)	0.51
Sex and neuter status						
Intact male (reference)	26 (21.7)	94 (78.3)	1.00	0.32	1.00	0.31
Male neutered	69 (24.2)	216 (75.8)	1.15 (0.69 to 1.93)	0.58	1.09 (0.65 to 1.84)	0.74
Intact female	15 (15.3)	83 (84.7)	0.65 (0.32 to 1.32)	0.23	0.60 (0.29 to 1.23)	0.16
Female neutered	77 (23.4)	252 (76.6)	1.10 (0.67 to 1.83)	0.67	1.02 (0.61 to 1.71)	0.94

OR Odds ratio, CI Confidence interval

Model: *N* = 832 old age dogs identified by one of five regular expressions (regex). Hosmer‐Lemeshow = 0.856. Adjusted model for age, old age regex, breed, sex and neuter status. The significance of P < 0.05 values are mentioned in bold.

**Table 3:** Musculoskeletal (M. skeletal) conditions. Univariable (unadjusted) and multivariable logistic regression analyses of factors associated with musculoskeletal modified ICD‐10 categorisation in the old age dog sample

Variable	M. skeletal disease present	M. skeletal disease absent	Unadjusted		Multivariable model	
	*N* (%)		OR (95% CI)	P	OR (95% CI)	P
Old age regex						
Ageing (reference)	52 (29.5)	124 (70.5)	1.00	0.65	1.00	0.69
Elderly	60 (36.6)	104 (63.4)	1.38 (0.87 to 2.17)	0.17	1.28 (0.80 to 2.06)	0.30
Geriatric	70 (35.5)	127 (64.5)	1.31 (0.85 to 2.03)	0.22	1.37 (0.88 to 2.15)	0.16
Old	35 (33.3)	70 (66.7)	1.19 (0.71 to 2.00)	0.51	1.19 (0.70 to 2.02)	0.52
Senior	61 (32.1)	130 (67.9)	1.13 (0.72 to 1.76)	0.30	1.31 (0.82 to 2.08)	0.25
Age	Median years					
	12.9	12.3	**1.07** (1.02 to 1.13)	**0.01**	**1.08** (1.02 to 1.15)	**0.01**
Breed	*N* (%)					
Mixed breed (reference)	72 (40.0)	108 (60.0)	**1.00**	**<0.01**	**1.00**	**<0.01**
Labrador retriever	48 (44.4)	60 (55.6)	**1.20** (0.74 to 1.94)	0.46	1.29 (0.79 to 2.11)	0.31
Jack Russell terrier	17 (28.3)	43 (71.7)	0.59 (0.31 to 1.12)	0.11	0.53 (0.28 to 1.02)	0.06
Cocker spaniel	9 (18.8)	39 (81.3)	**0.35** (0.16 to 0.76)	**0.01**	**0.36** (0.16 to 0.81)	**0.01**
Border collie	16 (34.0)	31 (66.0)	0.77 (0.39 to 1.52)	0.46	0.80 (0.40 to 1.58)	0.52
Springer spaniel	16 (44.4)	20 (55.6)	1.20 (0.58 to 2.47)	0.62	1.16 (0.55 to 2.41)	0.70
Other	100 (28.3)	253 (71.7)	**0.59** (0.41 to 0.86)	**0.01**	**0.61** (0.41 to 0.89)	**0.01**
Sex and neuter status						
Intact female (reference)	24 (24.5)	74 (75.5)	1.00	0.09	**1.00**	**0.04**
Intact male	43 (35.8)	77 (64.2)	1.72 (0.95 to 3.11)	0.07	1.76 (0.96 to 3.23)	0.07
Male neutered	107 (37.5)	178 (62.5)	1.85 (1.10 to 3.11)	0.02	**1.99** (1.17 to 3.39)	**0.01**
Female neutered	104 (31.6)	225 (68.4)	1.42 (0.85 to 2.39)	0.18	1.41 (0.83 to 2.38)	0.20

OR Odds ratio, CI Confidence interval

Model: *N* = 832 old age dogs identified by one of five regular expressions (regex). Hosmer‐Lemeshow = 0.025. Adjusted model for age, old age regex, breed, sex and neuter status. The significance of P < 0.05 values are mentioned in bold.

**Table 4:** Weight‐related conditions. Univariable (unadjusted) and multivariable logistic regression analyses of factors associated with the weight modified ICD‐10 categorisation in the old age dog sample

Variable	Weight issues present	Weight issues absent	Unadjusted		Multivariable model	
	*N* (%)		OR (95% CI)	P	OR (95% CI)	P
Old age regex						
Ageing (reference)	45 (25.6)	131 (74.4)	**1.00**	**<0.01**	**1.00**	**<0.01**
Elderly	49 (29.9)	115 (70.1)	1.24 (0.77 to 2.00)	0.37	1.43 (0.88 to 2.34)	0.15
Geriatric	64 (32.5)	133 (67.5)	1.40 (0.89 to 2.20)	0.14	1.40 (0.88 to 2.21)	0.15
Old	33 (31.4)	72 (68.6)	1.33 (0.78 to 2.27)	0.29	1.33 (0.78 to 2.29)	0.30
Senior	98 (51.6)	92 (48.4)	**3.10** (1.99 to 4.83)	**<0.01**	**2.90** (1.84 to 4.57)	**<0.01**
Age	Median years					
	12.0	12.8	**0.91** (0.86 to 0.96)	**<0.01**	**0.94** (0.88 to 0.99)	**0.02**
Breed	*N* (%)					
Mixed breed (reference)	65 (36.1)	115 (63.9)	1.00	0.51	1.00	0.62
Labrador retriever	38 (35.2)	70 (64.8)	0.96 (0.58 to 1.58)	0.87	0.964 (0.58 to 1.61)	0.89
Jack Russell terrier	16 (26.7)	44 (73.3)	0.64 (0.34 to 1.23)	0.18	0.71 (0.36 to 1.38)	0.31
Cocker spaniel	22 (45.8)	26 (54.2)	1.50 (0.79 to 2.85)	0.22	1.44 (0.74 to 2.82)	0.28
Border collie	17 (36.2)	30 (63.8)	1.00 (0.51 to 1.96)	0.99	1.04 (0.53 to 2.07)	0.90
Springer spaniel	10 (27.8)	26 (72.2)	0.68 (0.31 to 1.50)	0.34	0.68 (0.30 to 1.53)	0.35
Other	121 (34.3)	232 (65.7)	0.92 (0.63 to 1.34)	0.67	0.86 (0.58 to 1.27)	0.46
Sex and neuter status						
Intact male (reference)	44 (36.7)	76 (63.3)	1.00	0.36	1.00	0.36
Male neutered	91 (31.9)	194 (68.1)	0.81 (0.52 to 1.27)	0.36	0.75 (0.47 to 1.19)	0.23
Intact female	30 (30.6)	68 (69.4)	0.76 (0.43 to 1.34)	0.35	0.75 (0.42 to 1.35)	0.34
Female neutered	124 (37.7)	205 (62.3)	1.04 (0.68 to 1.61)	0.84	0.98 (0.63 to 1.54)	0.94

OR Odds ratio, CI Confidence interval

Model: *N* = 832 old age dogs identified by one of five regular expressions (regex). Hosmer‐Lemeshow = 0.870. Adjusted model for age, old age regex, breed, sex and neuter status. The significance of P < 0.05 values are mentioned in bold.

### Dental conditions

The odds of having a dental condition recorded increased by ~10% for every year of age [odds ratio (OR) 1.13, 95% confidence interval (CI) 1.07 to 1.21, P < 0.01], and cocker spaniels had ~2.7 times the odds of having a dental condition recorded (OR 2.71, 95% CI 1.38 to 5.31, P = <0.01) compared with the reference (mixed breed) (Table 1). The odds of having a dental condition recorded were greater for sexually intact female dogs (OR 1.64, 95% CI 1.15 to 2.34, P = 0.01), compared with sexually intact male dogs.

### Digestive conditions

There was no evidence of association between any signalment variables and the odds of having a digestive condition recorded. However, digestive conditions were more likely to be recorded in consultations identified by the “old,” “senior,” “geriatric” and “elderly” expressions compared with the “ageing” regex (Table 2; OR 1.86 to 3.74).

### Integumentary conditions

There was no evidence of an association between any of the predictor variables tested and the odds of having an integumentary condition recorded (Table S4).

### Musculoskeletal conditions

The odds of having a musculoskeletal condition recorded increased by ~8% for every year of age (OR = 1.08, 95% CI 1.02 to 1.15, P = 0.01), and dogs from both the cocker spaniel (OR = 0.36, 95% CI 0.16 to 0.81, P = 0.01) and other breed categories (OR = 0.61, 95% CI 0.41 to 0.89, P = 0.01) had lower odds compared with dogs in the mixed breed category. The odds of having a musculoskeletal condition recorded were greater in neutered males (OR = 1.99, 95% CI 1.17 to 3.39, P = 0.01) compared with sexually intact females (Table 3).

### Weight‐related conditions

There was a significant negative association between age and the odds of having a weight‐related condition recorded (OR = 0.94, 95% CI 0.88 to 0.99, P = 0.02). Multivariable analysis also suggested an association between the old age regex used and having a weight‐related condition recorded, whereby use of the term “senior” was associated with a greater odds (OR = 2.90, 95% CI 1.84 to 4.57, P < 0.01) compared with the reference term “ageing” (Table 4).

## DISCUSSION

Whilst veterinarians are often all too aware of the changing health and welfare challenges associated with caring for an old aged dog, definitions for old age are varied (Creevy et al., 2019; Davies, 2016; Fortney, 2012) and even arbitrary (Metzger, 2005). Attempts to better understand this life stage have been hampered by the difficulties of studying this population. To address this, we used a novel approach, leveraging a large dataset of EHRs, to identify a population of dogs specifically recorded by the attending veterinarian as being of old age. We suggest that this population can be used to explore the onset of old age and factors affecting it, as well as the most common conditions affecting OADs. We identified >2000 health conditions within clinical narratives from 832 old dogs, with a range of body systems included, with many dogs having several co‐morbidities involving different body systems. We suggest such a cohort of OADs is likely to be broadly equivalent to the geriatric life stage, where serious age‐related issues become more prevalent (Fortney, 2012; Metzger, 2005). It was unlikely for dogs <7.25 years to have one of the old age regexes recorded in their EHRs; therefore, it could be argued that this age could form the basis for a population‐level cut‐point for the transition between senior and geriatric life stages. Any preventive healthcare measures (e.g., health checks aimed at early identification of disease) should ideally be implemented before this age (Metzger, 2005). However, such a single cut‐point for the geriatric life stage is unlikely to be appropriate for all breeds of dog; indeed life tables usually show larger dogs becoming geriatric at an earlier age (Fortney, 2012; Metzger, 2005). Indeed, in the current study, breed differences were evident, with Jack Russell Terriers (median age 14.1 years) being significantly older than both Cocker Spaniels (11.7 years) and Labrador Retrievers (12.2 years) when considered to be of old age by their veterinarian. This older age of Jack Russells is consistent with findings of age at euthanasia in dogs (O'Neill et al., 2013), as well as a previous study in which Jack Russell terriers were shown to have longer life expectancy at 12.7 years (Teng et al., 2022). Using such health record data at scale may allow useful additional granularity to be added to age charts that are currently largely based only on a dog's weight (Fortney, 2012; Metzger, 2005).

In contrast to breed, neither sex nor neuter status affected the age at which the old age expressions were used. Other similar studies are lacking on the impact of sex on the transition to geriatric status. However, looking at end of life in dogs (either natural or euthanasia), Hoffman et al. (2018) suggested a complex picture mostly impacted by neuter status, with neutered animals surviving longer in both a referral population and a primary care dataset. In entire (non‐neutered) dogs, males lived longer than females. However, this was reversed in neutered animals, with females living longest, and in the primary care dataset, sex itself had no impact on longevity. Similarly, in their end‐of‐life study, McMillan et al. (2024) showed a significant female survival advantage. It seems likely that in veterinary visiting dogs, where most are euthanased rather than dying unassisted (Hoffman et al., 2018), decisions to end life will be heavily impacted by human behaviour in a similar way to other interventions like neutering, such that the older age of neutered dogs at death is more a reflection of owner choice, rather than inherent differences in the rate of underlying pathologies. Indeed it is clear that the timing of euthanasia in dogs is very much a negotiation between owners and their veterinary surgeons (Gray and Radford, 2022). Together, our results would suggest neutering does not impact the transition into geriatric life, rather the choice to end geriatric life through euthanasia; access to larger datasets is needed to further explore this.

The range and number of conditions affecting our cohort of OADs highlights the challenges that can be faced by primary care veterinarians when providing healthcare for geriatric dogs. Of course, some conditions were more common than others, with almost three quarters of all recorded complaints comprising the top five categories (weight‐related, integument, dental, musculoskeletal and digestive).

The importance of dental disease in this population is consistent with earlier work (Kortegaard et al., 2008; Wallis & Holcombe, 2020); even in our study population, which was pre‐selected for being of old age, the odds of having a dental condition recorded was still positively associated with the age of the dog. This finding emphasises the importance of managing dental disease proactively, so that its welfare impact might be mitigated. By preventing the formation of plaque and tartar, progression to periodontal disease can be avoided (Harvey et al., 2015), with the potential for improving overall health (Whyte et al., 2014) and preventing systemic disease (Rawlinson et al., 2011). The presence of tartar was common in the study population, being the most frequent sub‐category, affecting 109 of the 832 patients (13%), and comprising almost 5% of all recorded complaints. Compared with mixed breed dogs, cocker spaniels had >2.5 times the odds of dental disease being recorded, a finding consistent with previous research (O'Neill et al., 2014). The reasons for these associations are not known and would require further study. Nonetheless, knowledge of such breed predispositions can still be used when developing preventive healthcare strategies identifying at‐risk dogs (Metzger, 2005).

Compared with sexually intact male dogs, sexually intact female dogs had greater odds of having a dental condition recorded. Similar associations with dental disease severity and sex are well described in humans, where the increased risk of caries in females is attributed to hormonal affects, and the reduced volume and altered consistency of saliva (Lukacs & Largaespada, 2006). The reasons for the sex associations in dogs are not known but might be behavioural or hormonal in origin. Whatever the reasons for the associations between recording of dental conditions and the sex and neuter status of dogs, it could again be used to identify at‐risk individuals for preventive care.

Similar to dental disease, the prevalence of musculoskeletal disease increased with age, a finding consistent with a previous study (Anderson et al., 2018). Dogs from both the cocker spaniel breed and also those in the “Other breed” category had less than half the odds of having a musculoskeletal disease recorded compared with mixed breed dogs; this finding is again consistent with earlier studies in which Cocker Spaniels were found to have a lower prevalence of musculoskeletal issues when compared within the most popular breeds (O'Neill et al., 2014). Neutered males were at increased risk compared with intact females, again broadly consistent with earlier research showing male dogs and neutered dogs were 1.2 and 1.8 times more likely to experience osteoarthritis than female and entire dogs, respectively (Anderson et al., 2018). Suggested mechanisms for this male sex association for osteoarthritis include the differing sex hormone environment or body weight differences (Hays et al., 2007).

In contrast with previous research (Anderson et al., 2018), we found no association between the recording of a musculoskeletal condition and breeds such as Labrador retriever and springer spaniel. However, it is noteworthy that the most common subcategory, within musculoskeletal conditions, was “stiffness,” but this was reported in only a small proportion (16%) of records, and associations at the sub‐category level were unable to be investigated.

In future studies, leveraging regular expressions focusing on sub‐categories may allow more granular analysis of the impact of breed on age‐related conditions like stiffness.

In contrast to dental and musculoskeletal issues, no significant associations were identified for digestive, weight‐related and integument categories, perhaps, reflecting their variable nature; for example, the weight‐related condition category included both overweight, underweight, weight gain and weight loss subcategories. Using language processing methods, rather than simply reading clinical narratives might allow for the power of larger EHR datasets to be leveraged and the identification of more specific associations to an individual's breed and sex. Such an approach may be particularly useful for conditions that are readily identified and recorded in primary‐care veterinary practice such as stiffness, tartar, overweight and underweight.

As with any study, limitations should be considered. First, although using SAVSNET enables a large dataset to be studied, these data may not necessarily be representative of the entire UK canine population, because practices are not randomly recruited, and the database also only includes records for veterinary visiting dogs. Further, our analysis relied entirely on what veterinarians wrote in the EHRs, and this might be inaccurate or incomplete; for example, it is known that not everything discussed within a consultation is recorded within the EHR (Jones‐Diette et al., 2017). Our analyses also showed that recorded age is sometimes inaccurate. Any analyses predicated on text mining, even where regexes are used, remains susceptible to typographical errors and variations, and to practitioners simply not recording their observations; it is therefore likely that our estimate of the levels of old age should be considered an underestimate of the real situation.

A novel approach was used to suggest an overall age above which veterinarians are increasingly likely to identify old‐age‐related issues (7.25 years), and to identify common conditions recorded in such dogs. Our approach identified the main conditions and sub‐categories of disease seen in practice and showed some early indication of actionable breed and sex differences. Such an approach, particularly based on the terms ageing, geriatric and senior, could provide the novel scalable data source required for more tailored healthcare for OADs. This would also allow for linkage at scale to other relevant datasets derived by text mining and increasingly artificial intelligence that together could provide a more nuanced understanding of the ageing process experienced by individual animals and help target interventions to the right age for the right animals and the right diseases. These data have recently been used in the development of the PetSavers Ageing Canine Toolkit, which also included findings from a qualitative study with senior dog owners and veterinarians (Wallis et al., 2023) and quantitative surveys (Wallis et al., 2024).

### Author contributions

**J. Jackson:** Conceptualization (equal); data curation (lead); formal analysis (lead); methodology (equal); writing – original draft (equal); writing – review and editing (equal). **A. D. Radford:** Conceptualization (equal); data curation (lead); formal analysis (supporting); funding acquisition (supporting); investigation (supporting); methodology (equal); software (lead); supervision (equal); validation (equal); writing – original draft (equal); writing – review and editing (lead). **Z. Belshaw:** Conceptualization (supporting); formal analysis (supporting); funding acquisition (supporting); methodology (supporting); supervision (supporting); writing – original draft (equal); writing – review and editing (supporting). **L. J. Wallis:** Conceptualization (supporting); formal analysis (supporting); funding acquisition (supporting); methodology (supporting); supervision (supporting); writing – original draft (equal); writing – review and editing (supporting). **E. Kubinyi:** Conceptualization (supporting); formal analysis (supporting); funding acquisition (supporting); methodology (supporting); supervision (supporting); writing – original draft (supporting); writing – review and editing (supporting). **A. J. German:** Conceptualization (supporting); formal analysis (supporting); funding acquisition (supporting); methodology (supporting); supervision (supporting); writing – original draft (equal); writing – review and editing (supporting). **C. Westgarth:** Conceptualization (equal); data curation (supporting); formal analysis (equal); funding acquisition (lead); investigation (equal); methodology (supporting); supervision (lead); writing – original draft (equal); writing – review and editing (equal).

### Conflict of interest

AG is an employee of the University of Liverpool whose position is funded by Royal Canin and has also received financial remuneration and gifts for providing educational material, speaking at conferences, and consultancy work. CW has conducted consulting for MARS Petcare, Royal Canin, and Forthglade. ZB is part of EviVet Research Consultancy. The remaining authors declare that the research was conducted in the absence of any financial or personal relationship with other people or organisations that could influence or bias the content of the paper.

## Supporting information


**Table S1.** Regular expressions used to identify consultations containing terms old, ageing, geriatric, elderly and senior. Examples of accurate matches and identified inaccurate matches are indicated; any errors in text are those present in the original narrative. All text in SAVSNET is lowercased to avoid issues with case.
**Table S2.** Categories used to categorise consultation notes in this project (modified categories), and how they relate to the existing World Health Organization (WHO) International Statistical Classification of Diseases and Related Health Problems (ICD) ICD‐10 system (WHO, 2010).
**Table S3.** Frequency of modified ICD‐10 categories and associated most frequent sub‐categories identified in 832 old age dogs. Included are the two most common subcategories for each category, and any other sub‐categories present more than 30 times.
**Table S4.** Integumentary (integ) conditions. Univariable (unadjusted) and multivariable logistic regression analyses of factors associated with integument modified ICD‐10 categorisation in the OAD sample.

## Data Availability

The health record data that underpins this research can contain potential identifiers and so cannot be made publicly available. However, these data are available on reasonable request from savsnet@liverpool.ac.uk.
