# Supplementary material for: Using veterinary health records at scale to investigate ageing dogs and their common issues in primary care
Source: J Small Anim Pract. 2024 Dec 12;66(2):81–91. doi: 10.1111/jsap.13809 (PMC11821469; doi:10.1111/jsap.13809)
Supplement: Supplementary file 1 — Table S1. Regular expressions used to identify consultations containing terms old, ageing, geriatric, elderly and senior. Examples of accurate matches and identified inaccurate matches are indicated; any errors in text are those present in the original narrative. All text in SAVSNET is lowercased to avoid issues with case. Table S2. Categories used to categorise consultation notes in this project (modified categories), and how they relate to the existing World Health Organization (WHO) International Statistical Classification of Diseases and Related Health Problems (ICD) ICD‐10 system (WHO, 2010). Table S3. Frequency of modified ICD‐10 categories and associated most frequent sub‐categories identified in 832 old age dogs. Included are the two most common subcategories for each category, and any other sub‐categories present more than 30 times. Table S4. Integumentary (integ) conditions. Univariable (unadjusted) and multivariable logistic regression analyses of factors associated with integument modified ICD‐10 categorisation in the OAD sample. [file JSAP-66-81-s001.docx]

Supplementary Table 1

Regular expressions used to identify consultations containing terms old, ageing, geriatric, elderly and senior. Examples of accurate matches and identified inaccurate matches are indicated; any errors in text are those present in the original narrative. All text in SAVSNET is lowercased to avoid issues with case.

| Term | Regular expression | Accurate matches | Inaccurate matches |
| --- | --- | --- | --- |
| old | (?<!week\s)(?<!wk\s)(?<!weeks\s)(?<!ws\s)(?<!  wks\s)(?<!mo\s)(?<!m\s)(?<!mth\s)(?<!mths\s)(  ?<!month\s)(?<!months\s)(?<!day\s)(?<!days\s)  (?<!(\d)d\s)(?<!(\d)w\s)(?<!hrs\s)(?<![0-  4]\sy\s)(?<![0-4]\syr\s)(?<![0-4]\syear\s)(?<![0-  4]\syears\s)((?<![a-z])old\|(?<![a-z])ols\|(?<![a- z])older\|old age)(?!\s?(ulcer\|healing\|house\|people\|dirt\|fle a\|corneal\|scar\|food\|cruciate\|meloxaid\|injur\|wound\|scab\|uncle\|grandson\|daughter\|vet\|pr actice\|lesion\|superficial\|abscess\|fracture\|carri er\|sample\|cyst\|break\|passport\|trauma\|enoug h\|dried discharge\|damage\|collar\|rules\|laceration\|addr ess\|tick\|history\|healed\|resolving\|style\|child\| machine\|scratch\|one\|crust\|hotspot\|discharge  \|self\|selves\|staining\|clot\|diet\|prices\|baby\|ow ner\|keratitis\|scheme\|bandage\|chip\|bite\|xrays  \|ccl injury\|claw\|surgical site\|bumblefoot\|umbilical\|puncture\|blood\|se b\|dried\|coat\|fight\|adenoma\|pyoderma\|patch es\|number\|microchip\|saliva\|cut\|dry\|policy\|w art\|nail\|issue\|material\|carpet\|drip)) | result of old age is my suspicion.  owner wants to monitor as an old dog.  is older and slow  Advised old age is causing the problems but we still need to do something about it | Has older dog at home  Owner restarted old meds  Use an old sock  Some old discolorated wound |
| ageing | (?<!no\ssigns\sof\s)(?<!no\sevidence\sof\s)(?<! [a-z])ag(e)?ing\|age-related | suspect an ageing change, | Exercising less as other dog ageing |
|  |  | generally doing great for aging, slowing down v slightly | Vomiting aging yesterday |
|  |  | suspect age-related incontinence |  |
| geriatric | (geriatric\|geriatic\|geratric\|geriartic\|geriactric)(  ?!\s?feline\s?panel) | Geriatric dog  Bloods taken for geriatric screen  patient is geriatric. | attacks - nto typically like idiopatic or geriatric vestibular ttacs |
|  |  | as dog so geriatric and otherwise so well. |  |

| senior | (?<!o\s)(?<!owner\s)(?<!owner\sis\s)(?<!o\sis\s  )(senior\|senoir\|seniour\|(?<![a- z])snr\|seniro)(?!\s?(o(w\|\s\|,\|\.\|-  \|)\|lady\|gent\|man\|woman\|mother\|father\|rela tive\|neighbour\|parent\|couple\|vet\|nurse\|surg eon\|carer\|member\sof\sstaff)) | Suggested senior wellness profile, incase got underlying issues  Friendly dog. Healthy senior dog.  discussed pga bloods owner declined but will have drip as senior dog  advise more premium senior dog food | Snr died in he summer so just mum  Doesnt want to eat puppy food so is usigng senior; advised puppy is best but she does need some nutrition so sentior is better than nothing at all. |
| --- | --- | --- | --- |
| elderly | (?<!owner\swho\swas\s)(?<!o\s)(?<!owner\s)(?  <!owner\sis\s)(?<!o\sis\s)(eldery\|elderley\|elde rly)(?!\s?(o(w\|\s\|\,\|\.\|\-  \|\)\|lady\|gent\|man\|woman\|couple\|parents\|ne ighbour\|mother\|mum\|father\|relative\|client\|fr iend\|grandfather\|grandmother) | Overall for elderly dog doing really good.  dog v. distressed and elderly  Also as elderly patient is higher risk | communication difficult as owner deaf and elderly  rehomed to rspca from family where elderly member died |
|  |  | Dog elderly for his  type. Likely to spend more time lying down | better to do now on a slightly fitter 9 y/o than on a a more elderly 12 y/o |
|  |  |  | O does have another elderly dog who she has seen drinking more. |

Supplementary Table 2: Categories used to categorise consultation notes in this project (Modified categories), and how they relate to the existing World Health Organisation (WHO) International Statistical Classification of Diseases and Related Health Problems (ICD) ICD-10 system (WHO 2010).

| WHO ICD-10 classifiers | Modified Categories used in this project |
| --- | --- |
| Diseases of the ear and mastoid process | Auditory (middle, inner ear) |
| Diseases of the circulatory system Diseases of the respiratory system | Cardiopulmonary (coughing, sneezing, murmur, oedema) |
| Diseases of the digestive system | Dental |
| Diseases of the digestive system | Digestive (excluding teeth and anal glands; including lips as well as tongue to anus) |
| Endocrine, nutritional and metabolic diseases | Endocrine (including DM, Cushings, hypothyroidism etc.) |
| Diseases of the blood and blood-forming organs and certain disorders involving the immune mechanism | Immunological (including vaccine discussion) |
| Diseases of the skin and subcutaneous tissue | Integumentary (including external ear, otitis externa, nails and anal glands) |
| Diseases of the musculoskeletal system and connective tissue | Musculoskeletal (e.g. osteoarthritis, lameness) |
| Neoplasms | Neoplasia |
| Diseases of the nervous system | Neurological (including knuckling) |
| Diseases of the eye and adnexa | Ocular (including periocular skin, entropion/ectropion) |
| Certain infectious and parasitic diseases | Parasites (discussed or seen) |
| Diseases of the genitourinary system | Reproductive (include discussion of neutering) |
| Diseases of the genitourinary system | Urinary (infection, polyuria, incontinence) |
| Endocrine, nutritional and metabolic diseases | Weight |
| Clinical signs, and abnormal clinical and laboratory findings, not elsewhere classified | Other |
|  | Behaviour |
|  | Microchip (checked or given) |
|  | Euthanised |
|  | Travel |
|  | No Features Found |

Supplementary Table 3: Frequency of modified ICD-10 categories and associated most frequent sub-categories identified in 832 old age dogs. Included are the two most common subcategories for each category, and any other sub-categories present more than 30 times.

| Modified ICD-10 category | | Sub-category | | | | |
| --- | --- | --- | --- | --- | --- | --- |
| Name (number of patients affected: %) | Total issues in category | name | Rank | Number | % of category | % of all sub- categories (2,944) |
| Auditory (39: 4.7%) | 42 | Hearing loss | 15 | 38 | 90.5 | 1.3 |
|  |  | Causes of hearing loss – discussed |  | 2 | 4.8 | 0.1 |
| Behaviour (53: 6.4%) | 75 | Barking |  | 11 | 14.7 | 0.4 |
|  |  | Confused |  | 9 | 12 | 0.3 |
|  |  | Unsettled at night |  | 9 | 12 | 0.3 |
| Cardiopulmonary  (152: 18.3%) | 169 | Murmur | 5 | 57 | 33.7 | 1.9 |
|  |  | Coughing | 11 | 42 | 24.9 | 1.4 |
| Dental  (254: 30.5%) | 362 | Gingivitis | 19 | 32 | 8.8 | 1.1 |
|  |  | Tartar | 1 | 109 | 30.1 | 3.7 |
|  |  | Dental disease | 23 | 30 | 8.3 | 1.0 |
| Digestive  (187: 22.5%) | 230 | Vomiting | 14 | 39 | 17.0 | 1.3 |
|  |  | Inappetent | 17 | 32 | 13.9 | 1.1 |
| Endocrine  (7: 0.8%) | 8 | Hypothyroid |  | 3 | 37.5 | 0.1 |
|  |  | Insulin - discussion |  | 2 | 25 | 0.1 |
| Immunological (156: 18.8%) | 209 | Vaccination - L4 | 6 | 52 | 24.9 | 1.8 |
|  |  | Vaccination – KC | 18 | 32 | 15.3 | 1.1 |
|  |  | Vaccination | 3 | 66 | 31.6 | 2.2 |
| Integument  (235: 28.2%) | 270 | Nail - clipped |  | 28 | 10.4 | 1.0 |
|  |  | Anal glands |  | 25 | 9.3 | 0.8 |
| Microchip  (24: 2.9%) | 24 | Microchip - checked |  | 21 | 87.5 | 0.7 |
|  |  | Microchip - discuss |  | 2 | 8.3 | 0.1 |
| Musculoskeletal (278: 33.4%) | 375 | Stiffness | 4 | 60 | 16.0 | 2.0 |
|  |  | Lameness | 8 | 52 | 13.9 | 1.8 |
|  |  | Loss of muscle mass | 12 | 40 | 10.7 | 1.4 |
| Neoplasia  (164: 19.7%) | 200 | Mass/Lumps | 20 | 30 | 15.0 | 1.0 |
|  |  | Mass - mammary |  | 11 | 5.5 | 0.4 |
| Neurological  (93: 11.2%) | 107 | Proprioceptive deficit |  | 23 | 21.5 | 0.8 |
|  |  | Brain ageing |  | 19 | 11.7 | 0.6 |
| Ocular (163: 19.6%) | 205 | Cataracts | 7 | 53 | 25.9 | 1.8 |
|  |  | Sclerosis | 22 | 31 | 15.1 | 1.1 |
| Other (1: 0.1%) | 1 | Unknown |  | 1 | 100 | 0.0 |
| Parasites (14: 1.7%) | 14 | Fleas |  | 5 | 35.7 | 0.2 |
|  |  | Discuss anti-parasite treatment |  | 3 | 21.4 | 0.1 |
|  |  | Dispense anti-parasite treatment |  | 3 | 21.4 | 0.1 |
| Reproductive  (9: 1.1%) | 14 | Genital check |  | 4 | 28.6 | 0.1 |
|  |  | Pyometra - discuss |  | 3 | 21.4 | 0.1 |
| Urinary  (117: 14.1%) | 129 | Incontinence | 10 | 44 | 34.1 | 1.5 |
|  |  | Polyuria & polydipsia | 21 | 31 | 24.0 | 1.1 |
| Weight (289: 34.7%) | 457 | Ideal - BCS recorded | 9 | 47 | 10.3 | 1.6 |
|  |  | Diet – discussed | 13 | 24 | 5.3 | 0.8 |
|  |  | Weight loss | 2 | 93 | 20.4 | 3.2 |
|  |  | Weight gain | 16 | 35 | 7.7 | 1.2 |

496 Supplementary table 4: Integumentary (integ) conditions. Univariable (unadjusted) and multivariable logistic regression analyses of factors associated with integument modified ICD-10 categorisation in the OAD sample.

498

| **Variable** | **Integ. disease present** | **Integ. disease absent** | **Unadjusted** | | **Multivariable model** | |
| --- | --- | --- | --- | --- | --- | --- |
| *Old age regex* | N (%) | | OR (95% CI) | P | OR (95% CI) | P |
| Ageing **(Reference)** | 44 (25.0) | 132 (75.0) | 1.00 | 0.345 | 1.00 | 0.293 |
| Elderly | 43 (26.2) | 121 (73.8) | 1.066 (0.655-1.736) | 0.797 | 1.100 (0.667-1.815) | 0.709 |
| Geriatric | 62 (31.5) | 135 (68.5) | 1.378 (0.874-2.171) | 0.167 | 1.360 (0.858-2.155) | 0.191 |
| Old | 36 (34.3) | 69 (65.7) | 1.565 (0.923-2.654) | 0.096 | 1.562 (0.916-2.665) | 0.102 |
| Senior | 50 (26.3) | 140 (73.7) | 1.071 (0.670-1.714) | 0.773 | 0.975 (0.602-1.580) | 0.918 |
| *Age* | Median years | |  | | | |
|  | 12.2 | 12.6 | 0.968(0.917-1.021) | 0.233 | 0.966(0.910-1.025) | 0.249 |
| *Breed* | N (%) | |  | | | |
| Mixed Breed **(Reference)** | 49 (27.2) | 131 (72.8) | 1.00 | 0.573 | 1.00 | 0.574 |
| Labrador Retriever | 31 (28.7) | 77 (71.3) | 1.158 (0.682-1.965) | 0.587 | 1.153 (0.675-1.971) | 0.602 |
| Jack Russell Terrier | 25 (41.7) | 35 (58.3) | 1.087 (0.567-2.086) | 0.801 | 1.160 (0.600-2.242) | 0.660 |
| Cocker Spaniel | 23 (47.9) | 25 (52.1) | 1.021 (0.499-2.093) | 0.954 | 1.032 (0.498-2.137) | 0.932 |
| Border Collie | 13 (27.7) | 34 (72.3) | 0.651 (0.293-1.447) | 0.292 | 0.639 (0.287-1.426) | 0.275 |
| Springer Spaniel | 9 (25.7) | 26 (74.3) | 0.664 (0.273-1.615) | 0.366 | 0.660 (0.269-1.620) | 0.364 |
| Other | 104 (29.5) | 249 (70.5) | 1.228 (0.823-1.833) | 0.313 | 1.220 (0.812-1.834) | 0.337 |
| *Sex & Neuter Status* |  | | | | | |
| Intact Male **(Reference)** | 26 (21.7) | 94 (78.3) | 1.00 | 0.359 | 1.00 | 0.299 |
| Male Neutered | 83 (29.1) | 202 (70.9) | 1.486 (0.898-2.459) | 0.124 | 1.539 (0.924-2.562) | 0.098 |
| Intact Female | 27(27.6) | 71 (72.4) | 1.375 (0.739-2.557) | 0.315 | 1.393 (0.745-2.604) | 0.299 |
| Female Neutered | 99 (30.1) | 230 (69.9) | 1.556 (0.949-2.551) | 0.079 | 1.610 (0.976-2.655) | 0.062 |

499

500

501 Model: N = 832 old age dogs identified by one of five regular expressions (regex). Hosmer- Lemeshow= 0.531. Adjusted model for age, old age regex, breed, sex and neuter status. OR=odds ratio. CI = confidence interval.

504

505
